# Geographical and socioeconomic inequalities in the double burden of malnutrition among women in Southeast Asia: A population-based study

**DOI:** 10.1016/j.lansea.2022.04.003

**Published:** 2022-05-23

**Authors:** Tuhin Biswas, Nick Townsend, Ricardo Magalhaes, Md. Mehedi Hasan, Abdullah Al Mamun

**Affiliations:** a Institute for Social Science Research, The University of Queensland, Brisbane, Queensland, Australia; b ARC Centre of Excellence for Children and Families over the Life Course, The University of Queensland, Brisbane, Queensland, Australia; c Department for Health, University of Bath, Bath BA2 7AY, UK; d UQ Spatial Epidemiology Laboratory, School of Veterinary Science, University of Queensland, Gatton, Australia; e Children's Health and Environment Program, Child Health Research Centre, The University of Queensland, Brisbane, Queensland, Australia; f UQ Poche Centre for Indigenous Health,The University of Queensland, Brisbane, Queensland, Australia

**Keywords:** Geographical, Socioeconomic inequalities, Double burden of malnutrition

## Abstract

**Background:**

Countries in the South and Southeast Asia region grapple with significant challenges due to the double burden of malnutrition (DBM) in women. An understanding of the country specific DBM geographical and socioeconomic distribution in South and Southeast Asian countries will enable targeting of DBM interventions towards high-risk populations in the region. This study aimed to analyse anthropometric indicators for women's nutrition at national and subnational levels in seven South and Southeast Asian countries and assess the association between nutritional status and socioeconomic factors.

**Methods:**

We used population-representative cross-sectional data from the Demographic and Health Surveys conducted between 2000 and 2017, for seven South and Southeast Asian countries (Bangladesh, Cambodia, India, Myanmar, Nepal, Pakistan, and Timor-Leste) and estimated national and subnational prevalence of women underweight and overweight. Using a concentration index (CI), we measured relative and absolute inequality across underweight and overweight in urban and rural areas in these countries. In addition, we estimated the health achievement index, integrating mean coverage of nutritional status and the distribution of coverage by rural and urban populations.

**Findings:**

The prevalence of underweight women ranged from 7.0% (95% CI: 7.0-8.0%) in Pakistan in 2017 to 44.0% (95% CI: 42-45%) in Bangladesh in 2000 and overweight from 11.0% (95% CI: 10-12%) in Bangladesh in 2000 to 67.0% (95% CI: 66-68%) in Pakistan in 2017. In most countries, underweight disproportionately affected the poorest. The concentration indices for underweight were significant in all countries and ranged from –0.04 in Cambodia in 2014 to –0.38 in Pakistan in 2017. In contrast, overweight disproportionately affected the richest, with concentration indices for overweight significant in all countries, ranging from 0.16 in Cambodia in 2010 to 0.45 in Bangladesh in 2007. In most of the countries an absolute measure of inequality decreased overtime, whereas relative measures increased. Disachievement of underweight is more pronounced in rural populations compared to the urban ones.

**Interpretations:**

We noted large geographical and socioeconomic disparities in women DBM in South and Southeast Asian countries, at national and subnational levels. Planning, implementation, and evaluation of existing intervention programmes for food and nutrition should be based on subnational level needs and outcomes.

**Funding:**

This research is partially funded by the Australian Government through the Australian Research Council's Centre of Excellence for Children and Families over the Life Course (Project ID CE200100025).

**Research in context**

**Evidence before this study**

We systematically searched PubMed, EMBASE, and CINAHL with the following keywords: “Women,” “Female,” “Mother,” “underweight,” “chronic energy deficiency,” “overweight,” “obesity,” “body mass index,” “BMI,”, “socioeconomic inequalities”, “geographical variation”, “subnational level variation”, “South Asia,” “South East Asia,” as well as the names of individual countries. Literature search was conducted before July 30, 2020, to identify work about assessment of geographical and socioeconomic inequalities in the double burden of malnutrition among women in Southeast Asia. According to our inclusion criteria, one study in Pakistan assessed the geographical and socioeconomic inequalities in women and children's nutritional status in Pakistan in 2011. Nevertheless, none of the previous studies assessed the geographical and socioeconomic inequalities in the double burden of malnutrition among women in Southeast Asia using time rend data.

**Added value of this study**

There are few country specific studies but this is the first comprehensive study evaluating subnational level change in the prevalence of underweight, overweight, and socioeconomic inequalities among the women in South and Southeast Asian countries, based on nationally representative surveys. Recently, it was well documented that prevalence of underweight declined slowly and the dramatic increase of overweight in low-income and middle-income countries (LMIC). But there is little information about geographical variation over the time in South and Southeast Asian countries. This study also investigated the geographical variation and socioeconomic inequalities in the double burden of malnutrition within countries using different time point data. By use of concentration index (CI), we measured relative and absolute inequality across underweight and overweight in urban and rural areas in these countries. In addition, we estimated the health achievement index, integrating mean coverage and the distribution of coverage by rural and urban populations.

**Implications of all the available evidence**

At national and subnational levels among those countries with multiple time point data we found large geographical and socioeconomic disparities in women DBM. In addition, “disachievement” of women underweight is more pronounced in the rural population compared to the urban ones.

Alt-text: Unlabelled box

## Introduction

Most low-income and middle-income countries (LMIC) are affected by a double burden of malnutrition (DBM), defined by the co-existence of underweight and overweight in an individual, household and population.^1^ A recent WHO series on the Double Burden of Malnutrition reported that DBM has increased in many Asian and sub-Saharan African countries.^1^ The DBM is of particular concern among women of reproductive age because it imparts multiple long-term adverse health consequences to individuals, societies, and health care systems.^2^, ^3^, ^4^ Undernutrition in women is associated with adverse pregnancy outcomes including maternal mortality, delivery complications, preterm birth and intrauterine growth retardation.^5^ Conversely maternal obesity leads to several adverse maternal and fetal complications during pregnancy, delivery, and post-partum.^6^ Nutrion in women is obvioulsy important for women's health and that for the future generation.^7^

A recent study conducted in eight South and Southeast Asian countries reported that the average annual rate of increase of women overweight (8.4%) for the period 1996 to 2016 was higher than the average annual rate of reduction for women underweight (1.3%).^8^ However, although data on women's nutrition have been reported at the national level for South and Southeast Asian countries^9^, ^10^, ^11^ little information on within-country geographical variation is available. From a programmatic perspective, identification of geographical disparities can assist the deployment of interventions to those communities most in need.

A systematic review and meta-analysis on this region reported that underweight was more prevalent in rural areas, among women of the youngest age group (15–19 y), and among those with no education. In contrast, overweight was higher in urban areas, among women of older age, and among those with higher education.^12^ Another recent global study also reported that prevalence of underweight declined slowly and the dramatic increase of overweight in LMICs.^13^ Furthermore, a recent study reported that DBM has increased in the poorest in low income countries, mainly due to increases in overweight and obesity.^14^ Disparities in the nutrition related outcomes between the poor and the rich are not new,^15^^,^^16^ although in South and Southeast Asia the available literature documenting socioeconomic inequality in DBM has mainly focused on individual countries.^17^^,^^18^ Hence, within country geographical variation of socioeconomic inequalities in women DBM remain poorly understood. This is important as within country nutritional status may be different from those found between countries, with improvements in population micronutrient intake and reductions in early life infections occurring in recent decades^4^^,^^19^^,^^20^ It is important to know the subnational level distribution of DBM if effective population level interventions are to be developed and implemented. Furthermore, a lack of understanding of the within country DBM geographical and socioeconomic distribution in South and Southeast Asian countries is a barrier to tackling the problem within the region.

The overall aim of this study was to assess (i) the prevalence of women underweight and overweight/obesity at the national and subnational levels in South and Southeast Asia, and (ii) to quantify the socioeconomic inequality in DBM (women underweight and overweight/obesity) within and between countries in the region.

## Methods

### Data sources and procedures

This study used large-scale, nationally representative data from seven South and Southeast Asian countries, namely Bangladesh, Cambodia, India, Myanmar, Nepal, Pakistan, and Timor-Leste conducted between 2001 and 2017 under the Demographic and Health Surveys (DHS) program.^21^ The surveys have been designed to be nationally representative and cross-sectional data on population, health, reproduction, and nutrition have been gathered in more than 300 surveys in over 90 countries to date. This study applied in total 18 surveys from seven counties in South and Southeast Asia conducted in different time points. In the DHS, households were randomly selected based on probability sampling. From the household, women aged between 15 and 49 years were identified for a further in-depth interview. The DHS survey methodology and questionnaire were reviewed and approved by the ICF Institutional Review Board. Informed consent was collected from all participants prior to data collection with anonymized data being made publicly available. In addition, each survey was approved by the country specific ethics committee. The protocol of this project also approved by The University of Queensland, Ethics Review Board.

### Measurement of double burden of malnutrition

In all DHS surveys, trained personnel collected height and weight measurements following a standardized procedure. Weight was measured with solar-powered scales accurate to 0.1 kg and height was measured with standardized measuring boards accurate to 0.1 cm. BMI was calculated as weight (kg)/height (m^2^). Asian-specific BMI cutoffs were used to define underweight as <18.5kg/m^2^ and overweight and obese (higher BMI) as ≥23kg/m^2^.^22^, ^23^, ^24^, ^25^ As the prevalence of obesity was low in some countries, the categories of overweight and obesity were combined to be presented as overweight/obesity. Prevalence of underweight and overweight/obesity were calculated through weighting collected data to ensure samples were representative of the adolescent population of the country they were collected in. This involved using strata and primary sampling units at the country level. Weighted prevalence with corresponding 95% confidence intervals (95%) CIs were then reported by country. The overall pooled estimate of underweight and overweight/obesity was generated for individual countries to allow cross-country comparisons. Pooled prevalence, with associated 95% CIs, were also presented. Heterogeneity was examined using the I^2^-statistic and a high level of inconsistency (I^2^ > 50%) warranted the use of a random effect assumption in analysis. Then we assessed the country specific trend of women underweight and overweight/obesity over the year.

### Measurement of socioeconomic status

In DHS, wealth index was used to measure the household socioeconomic status. The wealth index is a composite score calculated by the DHS staff from principal component analysis on routinely collected data on a household's ownership of selected assets, including televisions, bicycles, cars, materials used for housing construction, types of water access, sanitation facilities, and types of fuel used. This continuous scale of relative wealth was then categorized into quintiles: Lowest (quintile 1, Q1), second (quintile 2, Q2), middle (quintile 3, Q3), Fourth (quintile 4, Q4), and Highest (quintile 5, Q5).

### Measurement of inequality

We estimated the concentration index (CI), along with standard error (SE) to measure the inequality in underweight and overweight/obesity prevalence across different socioeconomic groups of households, as proposed by Wagstaff.^26^ In calculating CI, we ranked households according to their socioeconomic characteristics from the poorest to the richest. The index is bounded between -1 and +1. A CI value of zero means that no SES-related inequality exists.^27^, ^28^, ^29^ When applying to the variables of interest for this study a positive value would imply a higher prevalence of either underweight or overweight/obesity in the more affluent, or high socioeconomic populations, whilst a negative value would imply that prevalence is more concentrated among less affluent groups.^28^, ^29^, ^30^ In addition to CI we also calculated a wealth index by comparing the poorest to the richest quintiles for each variable. In doing so we calculated an absolute measure of inequality (Q1-Q5) and a relative measure (Q1/Q5). Using the rate ratio as measure, relative inequalities tend to be larger at lower overall levels. Using the rate difference as measure, absolute inequalities tend to be low at both very low and very high overall levels. The absolute difference suggests that there were similar changes in both Q1 and Q5 over time, the relative change suggest that these were greatest in more affluent increasing the relative difference. Negative CI values indicate an increasing concentration of the variable of interest among the poor, whilst a positive value indicates the variable of interest is disproportionately present among the rich group.

### Achievement index

The health achievement index has been calculated for the socioeconomic distribution of all indicators using the measure of “achievement” as proposed by Wagstaff.^32^ It was shown that by measuring achievement as a weighted average of health levels, where the weights are the same as used in the extended concentration index, the resultant index is in fact equal to the product of the average and the complement of the extended concentration index. A larger value of the index is considered as higher health disachievement to one group of the population in comparison to others, by combining inequality with average level of health.^32^ In general, rising average values mean that the level of “disachievement” becomes larger and deceasing average values mean that the level of “disachievement” becomes smaller. We assessed achievement and disachievement index over the time.

In national and subnational level analysis, the complex sampling design with weighted sample was adjusted in all analyses and variations in errors by using “svy” command in STATA. We used STATA (version 13) to perform all analyses.^31^ District boundaries were georeferenced and linked to the district DBM notification rate per year and choropleth maps were developed for visualization using a geographic information system (ArcGIS Desktop version 10.3.1; ESRI, Redlands, CA, USA). We used country specific place of residence (as rural and urban) information.

## Role of the funding source

The funder had no role in the design of the study, data collection, analysis or interpretation of the results, and the content of the manuscript was written independently of the funder. The corresponding author had full access to all the data and all authors agreed with the decision to submit for publication.

## Results

Data from 933,836 women from seven countries in South and Southeast Asia were analyzed in this study (Table 1). The majority of the sample came from India (n = 699,686), followed by Bangladesh (n = 68,685), and Nepal (n = 45,055). The highest proportion of women in the richest quintile (29.7%) was found in Cambodia in 2014, and the lowest was found in Bangladesh in 2007 (16.1%).

**Table 1:** General characteristics of the study population.

Country	Year	Total sample, n	Mean age ± SD, y	Lowest household wealth quintile, %	Highest household wealth quintile, %
Bangladesh	2014	17,863	31.0 ± 9.2	18.2	21.6
Bangladesh	2011	17,842	30.8 ± 9.2	17.4	23.5
Bangladesh	2007	10,996	30.6 ± 9.3	16.1	26.7
Bangladesh	2004	11,440	30.0 ± 9.4	17.9	25.5
Bangladesh	2000	10,544	29.7 ± 9.3	18.2	25.3
Nepal	2016	53,848	29.4 ± 9.5	21.2	17.8
Nepal	2011	12,674	28.7 ± 9.6	19.3	24.3
Nepal	2006	10,793	28.6 ± 9.7	20.5	21.7
Nepal	2001	8726	30.8 ± 9.0	22.5	21.3
India	2016	699,686	29.8 ± 9.7	19.0	18.8
Myanmar	2016	12,885	31.6 ± 9.8	18.4	20.9
Pakistan	2017	15,068	32.3 ± 8.3	19.2	20.6
Pakistan	2013	13,558	32.6 ± 8.5	18.3	23.9
Timor Leste	2016	12,607	28.7 ± 10.0	16.3	21.3
Timor Leste	2009	13,137	28.6 ± 10.0	19.4	19.0
Cambodia	2014	17,578	30.1 ± 9.8	17.4	29.7
Cambodia	2014	18,754	29.5 ± 10.1	17.4	28.6
Cambodia	2005	16,823	29.6 ± 10.2	19.4	23.0
Pooled		933,836	29.9 ± 9.7	18.91	19.93

### Geographic variation in the prevalence of women underweight and overweight

**Figure 1:**
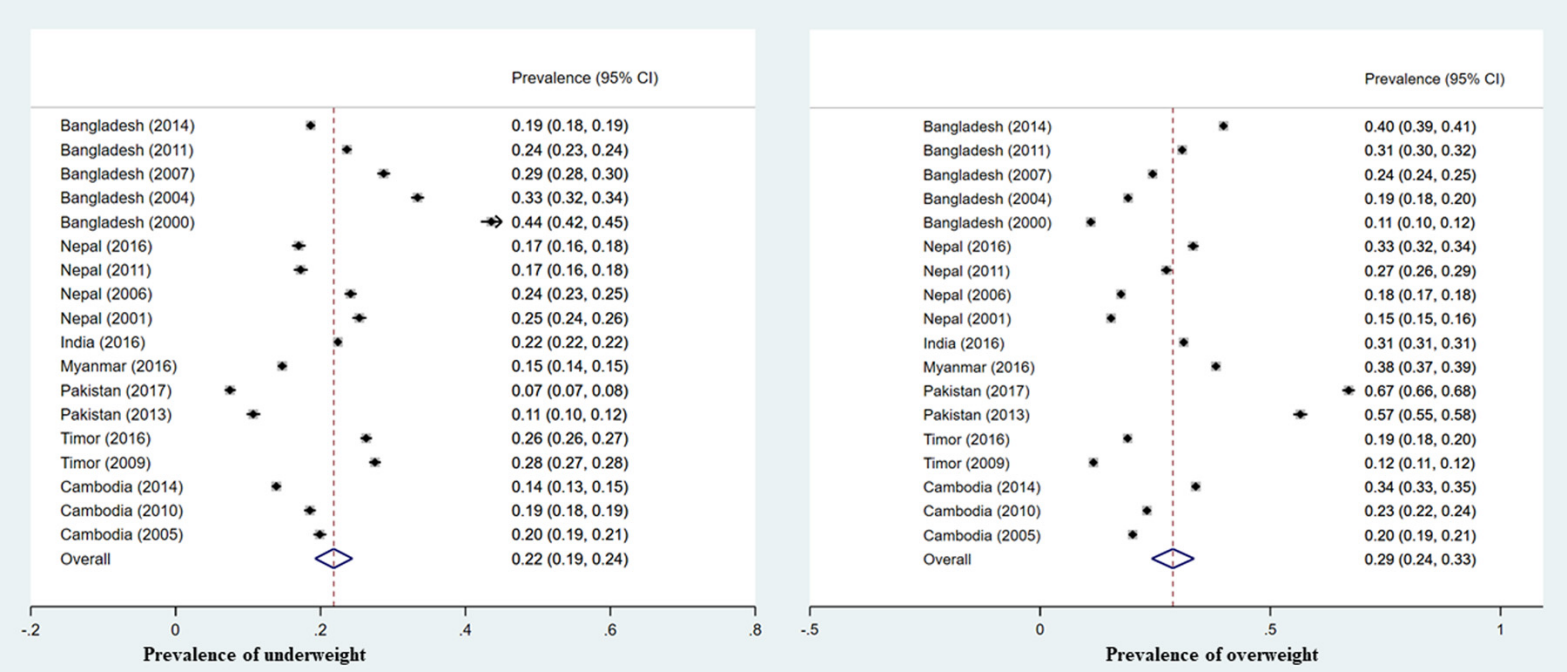
Underweight and overweight/obesity trends in south and Southeast Asia.

The pooled prevalence of women underweight and overweight in the South and Southeast Asian region was 22% (95% CI: 19-24%) and 29 % (95 CI: 25-33%) respectively (Figure 1) with variation found by country and year for both. Women underweight ranged from 7.0% (95% CI: 7.0-8.0%) in Pakistan in 2017 to 44% (95% CI: 42-45%) in Bangladesh in 2000. Women overweight ranged from 11% (95% CI: 10-12%) in Bangladesh in 2000 to 67% (95% CI: 66-68%) in Pakistan in 2017. The highest prevalence of underweight was observed in Bangladesh in 2000 (44%) and overweight/obesity observed in Pakistan in 2017 (67%) (Figure 1). Decreasing trends in underweight and increasing trends in overweight prevalence over this period were also found within countries for which numerous time point data were available, including Bangladesh, India, Nepal, Timor-Leste, and Cambodia (Figure 1). For example, in Bangladesh in 2000 the prevalence of women underweight was 44%, with this decreasing to 19% in 2014 (Figure 1). Conversely, the overweight/obesity prevalence of women in Bangladesh was 11% in 2004, with this increasing to 40% in 2014.

Subnational level prevalence of underweight and overweight for all countries are presented in supplementary figures. In all years for which data were available for Nepal, the prevalence of underweight was higher in the Far-western region and lowest in the Western region (p=<0.005). Prevalence of overweight/obesity was higher in the Western region in all years, rising from 19% in 2001 to 42% in 2016. Underweight prevalence in India's states ranged from 6% in Sikkim to nearly one-third (32%) in Jharkhand. Whereas overweight ranged from 19% in Jharkhand to 56% in Lakshadweep (supplementary figure). In Cambodia, the highest prevalence of underweight was observed in year 2005 (25%) was found in Takeo state and 30% in 2014 Kampong state. For overweight the highest prevalence was observed in Koh Kong state (31%) in year 2005 and 48% in Pailin state in year 2014 . In Pakistan in all states, the prevalence of overweight/obesity was found to be more than 60% (supplementary figure). In Bangladesh, in 2000 the highest prevalence of underweight was observed in Sylhet division (55%) which was remain highest in 2014 (30%). For overweight from 2004 (19%) overweight was more prevalent in Khulna divison in 2014 (45%). In Timor-Leste, in 2009 the highest prevalence of underweight was observed in Oecussi region (35%) which was highest in 2016 (37%). On the other hand, highest prevalence of overweight/obesity was observed in Covalima region in 2009 (24%) which was remain highest in 2016 (27%). In Maynamar highest prevalence of underweight and overweight/obesity was observed in Rakhine region (20%) and Sagaing region (42%) respectively.

### Socioeconomic inequality

In almost all countries, underweight disproportionately affected the poorest. The concentration indices for underweight were significant in all countries and ranged from –0.04 in Cambodia in 2014 to –0.38 in Pakistan in 2017. Conversely, overweight disproportionately affected the richest with concentration indices for overweight/obesity significant in all countries, ranging from 0.16 in Cambodia in 2010 to 0.45 in Bangladesh in 2007 (Table 2.1, 2.2). Prevalence measures also demonstrated that underweight was more prevalent and overweight less prevalent amongst individuals in the poorer quintiles in comparison to those in the more affluent quintiles. Over time in almost all countries, the absolute difference between the poorest and most affluent quintiles (Q1-Q5) decreased whilst the relative difference (Q1/Q5) increased. For example, in Bangladesh, the absolute difference in underweight prevalence reduced from 39% in 2004 to 25% in 2014, whereas over the same period, the absolute measure of inequality increased from 2.9 in 2004 to 4.5 in 2014. It means, there were similar changes in both Q1 and Q5 over time, the relative change suggest that these were greatest in more affluent increasing the relative difference.

**Table 2.1:** Estimated prevalence of underweight by socioeconomic status (SES) quintile, in South and Southeast Asia.

	Country	SES quintiles					Absolute difference	Relative difference	Concentration Index (CI)	SE
		Q1	Q2	Q3	Q4	Q5	Q1-Q5	Q1/Q5		
**Underweight**	Bangladesh (2014)	31.79	25.20	19.23	12.94	7.07	24.72	4.50	-0.33	0.02
	Bangladesh (2011)	40.20	30.18	25.36	19.62	8.71	31.49	4.62	-0.32	0.01
	Bangladesh (2007)	44.11	36.28	33.05	26.50	12.96	31.15	3.40	-0.27	0.02
	Bangladesh (2004)	47.61	41.66	36.67	31.79	16.55	31.06	2.88	-0.24	0.01
	Bangladesh (2000)	58.88	51.76	46.3	41.32	19.86	39.02	2.9	-0.27	0.01
	Nepal (2016)	18.87	19.84	19.82	16.12	9.10	9.77	1.93	-0.15	0.03
	Nepal (2011)	21.14	20.11	18.60	15.88	12.25	8.89	2.38	-0.14	0.03
	Nepal (2006)	24.27	31.45	27.65	24.20	15.08	9.19	2.64	-0.16	0.03
	Nepal (2001)	25.65	28.60	31.93	27.72	14.85	10.80	2.38	-0.10	0.02
	India (2016)	34.67	27.50	21.51	16.55	11.47	23.20	3.02	-0.27	0.00
	Myanmar (2016)	19.29	14.57	14.89	13.13	12.33	6.96	1.56	-0.11	0.02
	Pakistan (2017)	16.02	7.57	7.43	4.39	3.26	12.76	4.91	-0.38	0.05
	Pakistan (2013)	18.18	12.45	13.39	7.73	4.14	14.04	4.39	-0.36	0.05
	Timor Leste (2016)	31.54	28.61	25.42	23.70	24.02	7.52	1.31	-0.08	0.02
	Timor Leste (2009)	29.73	29.02	28.07	28.40	22.07	7.66	1.35	-0.07	0.02
	Cambodia (2014)	16.82	14.12	14.26	13.40	12.21	4.61	1.38	-0.04	0.02
	Cambodia (2010)	22.92	20.85	17.85	17.85	15.45	7.47	1.48	-0.08	0.02
	Cambodia (2005)	23.99	22.27	22.45	17.74	14.39	9.60	1.67	-0.10	0.02

**The concentration index (CI), along with standard error (SE) to measure the inequality in underweight and overweight/obesity prevalence across different socioeconomic groups of households.

**Table 2.2:** Estimated prevalence overweight/obesity by socioeconomic status (SES) quintile, in South and Southeast Asia.

	Country	SES quintiles					Absolute difference	Relative difference	Concentration Index (CI)	SE
		Q1	Q2	Q3	Q4	Q5	Q1-Q5	Q1/Q5		
**Overweight**	Bangladesh (2014)	19.35	26.63	35.05	46.85	65.74	-46.39	0.29	0.38	0.01
	Bangladesh (2011)	11.00	16.76	24.78	35.59	35.59	-24.59	0.31	0.42	0.01
	Bangladesh (2007)	7.62	10.94	14.96	24.84	49.94	-42.32	0.15	0.45	0.02
	Bangladesh (2004)	5.42	7.06	12.12	19.60	41.74	-36.32	0.13	0.45	0.02
	Bangladesh (2000)	1.42	4.21	5.56	8.95	33.4	-31.98	0.04	0.57	0.04
	Nepal (2016)	21.82	28.05	27.67	35.90	56.29	-34.47	0.39	0.27	0.02
	Nepal (2011)	11.95	11.95	20.74	20.74	20.74	-8.79	0.58	0.37	0.02
	Nepal (2006)	9.07	9.51	12.20	17.91	35.96	-26.89	0.25	0.36	0.03
	Nepal (2001)	7.95	8.01	10.31	12.71	35.65	-27.70	0.22	0.37	0.03
	India (2016)	12.93	21.31	30.75	41.08	50.68	-37.75	0.26	0.35	0.00
	Myanmar (2016)	24.06	32.78	38.42	43.32	49.61	-25.55	0.48	0.20	0.02
	Pakistan (2017)	43.00	61.25	68.47	75.80	83.42	-40.42	0.52	0.37	0.03
	Pakistan (2013)	36.36	46.21	54.99	65.76	73.06	-36.70	0.50	0.38	0.02
	Timor Leste (2016)	12.04	13.00	17.12	22.46	27.38	-15.34	0.44	0.21	0.02
	Timor Leste (2009)	6.51	8.01	8.99	13.15	21.44	-14.93	0.30	0.28	0.02
	Cambodia (2014)	24.99	30.46	32.60	36.29	39.68	-14.69	0.63	0.11	0.02
	Cambodia (2010)	14.01	18.35	19.89	26.38	31.09	-17.08	0.45	0.16	0.02
	Cambodia (2005)	10.81	13.24	15.12	23.97	33.87	-23.06	0.32	0.27	0.02

**The concentration index (CI), along with standard error (SE) to measure the inequality in underweight and overweight/obesity prevalence across different socioeconomic groups of households.

Table 3 presents the health achievement index for underweight and overweight/obesity by urban and rural residence. In most of the countries, the average level of underweight was higher in rural areas compared to urban areas. This “disachievement” of underweight is more pronounced in the rural population compared to the urban ones. In India, the prevalence of underweight is greater in rural compared to urban areas and although the level of inequality within rural areas are lower the overall HAI is higher, meaning that there is a greater health disachievement, or lower health achievement, in rural areas (21.7 vs 23.6). In contrast to underweight in most of the countries, the average level of overweight was higher in urban areas compared to rural areas. This means “disachievement” of women overweight is more pronounced in the urban population compared to the rural ones. For example, in Pakistan over the year, the prevalence of overweight is greater in urban compared to rural area. It means urban areas had lower health achievement compared to rural area.

**Table 3:** Place of residence specific estimated underweight and overweight/obesity rates by place of residence, in South and Southeast Asia.

Country	Total (underweight)		Urban underweight		Rural underweight		Total (overweight)		Urban overweight		Rural overweight	
	Coverage	HAI	Coverage	HAI	Coverage	HAI	Coverage	HAI	Coverage	HAI	Coverage	HAI
Bangladesh (2014)	19.25	25.63	18.06	22.21	19.89	24.46	38.72	23.82	28.17	21.85	34.15	26.48
Bangladesh (2011)	24.81	32.81	22.48	27.59	25.75	31.61	24.74	14.40	31.31	25.42	15.13	12.28
Bangladesh (2007)	30.58	38.87	28.29	35.92	31.49	39.98	21.66	11.83	23.69	16.67	17.50	12.32
Bangladesh (2004)	34.86	43.39	33.95	42.33	36.16	45.07	17.19	9.42	7.22	4.74	14.48	9.51
Bangladesh (2000)	43.62	55.64	40.03	53.34	45.09	60.07	10.70	4.53	13.60	8.10	8.21	4.90
Nepal (2016)	16.75	19.24	16.83	17.44	17.58	18.22	33.95	24.62	34.11	34.37	32.15	32.39
Nepal (2011)	17.60	19.99	18.42	18.92	17.39	17.86	17.22	10.86	27.68	29.13	25.65	27.00
Nepal (2006)	24.53	28.47	27.83	30.95	23.98	26.67	16.93	10.82	15.98	16.19	15.35	15.54
Nepal (2001)	25.75	28.33	32.81	36.72	25.71	28.77	14.93	9.36	11.32	9.50	13.98	11.74
India (2016)	22.34	28.41	21.05	21.79	22.82	23.63	31.35	20.26	34.92	34.76	29.86	29.72
Myanmar (2016)	14.84	16.43	15.29	14.75	15.41	14.86	37.64	30.13	40.90	43.44	35.96	38.20
Pakistan (2017)	7.73	10.69	6.73	7.68	8.00	9.12	66.39	41.56	68.44	64.49	64.68	60.94
Pakistan (2013)	11.18	15.17	7.68	8.60	11.68	13.08	55.28	34.06	59.96	57.92	54.09	52.26
Timor (2016)	26.66	28.76	25.57	24.44	26.90	25.70	18.40	14.62	20.61	21.62	17.58	18.44
Timor (2009)	27.46	29.51	28.77	28.61	26.98	26.83	11.62	8.39	12.62	12.75	11.20	11.31
Cambodia (2014)	14.16	14.78	14.15	11.74	14.15	11.74	32.80	29.24	35.72	42.69	33.38	39.89
Cambodia (2010)	18.98	20.50	19.33	16.49	19.33	16.49	21.94	18.36	25.98	31.72	21.25	25.95
Cambodia (2005)	20.17	22.18	20.62	19.03	20.62	19.03	19.40	14.10	19.18	20.00	19.60	20.44

* Coverage-average of wealth index specific prevalence; HAI – Health achievement index

Country specific variations of socioeconomic inequality at subnational level are presented in supplementary figures*.* In Bangladesh, divisional specific analysis showed that the absolute measure of inequality for underweight was highest in the Sylhet division in all years but reduced from 44.5% in 2004 to 33.1% in 2014 (supplementary figure). In Timor-Leste absolute measure of inequality for underweight constant in Ermera region over the year, it was 30.4% in 2009 and 24.6% in 2016. Conversely, the absolute measure of inequality for overweight was higher in Ainaro region in 2009 (-23.28%) and Manatuto region in 2016 (-29.4%). Implying that prevalence of overweight was greater among richest women and higher in Ainaro region in 2009 and Manatuto region in 2016. In India, health achievement index was higher in Gujarat state (34.42) and lower in Manipur state (9.32). For overweight, the index was higher in Puducherry state (50.52) and lower in Jammu and Kashmir state (22.36) (supplementary figure).

## Discussion

Our study contributes to the current discussion on DBM in women in South and Southeast Asian countries in several ways. First, our results provide an evidence base on women nutritional status (underweight and overweight) at national and subnational levels and describes the relationship with socioeconomic factors. The quality of evidence provided by our approach is underpinned by the use of the nationally representative DHS data from seven South and Southeast Asian countries. The comprehensiveness of our dataset for analysis enables a full understanding of the extent of geographical and socioeconomic inequalities in DBM in South and Southeast Asian countries. Secondly, the focus on trend analysis of women nutritional status and socioeconomic inequality in DBM provides temporal insights into within country geographical regions which have been consistently more vulnerable over time.

The results of this study indicate that among South and Southeast Asian women overweight/obesity increased gradually overtime and now exceeds the prevalence of underweight in almost all countries. In all countries we found geographical variation of underweight and overweight/obesity at subnational level. Aligned with our findings, other studies have also reported wide variation in the prevalence of underweight and overweight/obesity at national and regional levels.^33^^,^^34^ A recent study also reporting that if recent trends in mean BMI continue,^35^ the prevalence of obesity will continue to rise at the recent alarmingly high rates.

The authors found few studies that described socioeconomic inequalities of malnutrition in South and Southeast Asia.^17^^,^^18^ This included a study from Pakistan reporting large social and geographical inequalities in child and maternal nutrition, that were masked by national and provincial averages.^15^ Our results demonstrate that in almost all countries, underweight disproportionately affected the poorest. Conversely, overweight was a greater problem in the more affluent, with concentration indices for both underweight and overweight significant in all countries. According to our results, we found wealth related inequalities in country specific prevalence of women underweight and overweight/obesity. Our study also recommends nutrition specific programs for poorest groups. In terms of programme coverage, there is an urgent need to cover vulnerable subnational areas, such as mass population coverage, a broad strengthening of the whole system, either alone or combined with targeting, is required.^15^ In this respect, monitoring the distribution of DBM at a subnational level across different socioeconomic groups and regions can provide a useful tool for health policy-makers.

The present study is the first to report national and subnational trends of women nutritional status and socioeconomic inequality in DBM. While our analyses utilize comprehensive population-based nationally representative samples covering both rural and urban areas of seven South and Southeast Asian countries our results should be interpreted in mind of a few limitations. Firstly, we mainly focused on population level DBM, at individual and household levels. All data were cross-sectional in nature and collected from countries in various years, which limits our ability to make comparisons between countries. This highlights the need for consistent and regular data collection in all countries, to measure the progress of DBM. Secondly, all data were cross-sectional in nature at different time points, which limits our ability to argue the casual directions of the associations we observed and comparison across countries. We recommended consistent and regular data collection in all countries to measure the progress of DBM. However, repeated cross-sectional data are useful for investigating population-level trends in prevalence, which was the main aim of the study. In addition, heterogeneity was found in both the years of available data between countries and in the sample characteristics. Thirdly, which is common in data collection of this type, is that certain demographic information is self-reported. Although this could introduce some bias, all height and weight measurements were collected by trained staff following standardized procedures, ensuring none were self-reported, thereby avoiding bias in the outcome measures. Fourthly, SES was calculated from wealth index and not from household income. But wealth-based SES is already well established and widely used. Finally, we were not able to examine intra individual occurrence and determinants of malnutrition, as the data we used were cross-sectional in nature. Future work using longitudinal or cohort data could be used to examine this intra individual occurrence of DBM and its determinants. Changes in sample characteristics may reflect population changes in these countries, such as the increasing proportion of the sample living in urban areas over time. To counter this, some analyses were stratified by these characteristics, and they were controlled for in regression analyses.

## Conclusion

In conclusion, the results of this study indicate that not only the degree of socioeconomic inequality in DBM but also its pattern should be of concern in setting health policies. Our analysis provides further evidence from the subnational level in seven South and Southeast Asian countries that concerted action on subnational level nutrition interventions is needed urgently. Nutrition monitoring with an equity lens should become an integral component of tracking progress towards optimal nutrition at a population level. In addition to that, subnational level effective strategic and nutrition programs need to be addressed to combat the DBM in South and Southeast Asian countries.

## Contributors

TB contributed towards literature search, data analysis and interpretation, figures and tables, and writing of the manuscript. NT contributed towards the drafting of the protocol, review of the study design, data collection and interpretation and provided a critical review of the manuscript. RSM contributed towards data analysis plan and provided oversight and interpretation of the analyses. MMH contributed towards the study design, protocol writing, and data interpretation. AM contributed towards the design of the manuscript, development of the protocol, and critical evaluation and interpretation of the results and critical review of the manuscript.

## Declaration of interests

All other authors declare no competing interests.

## Data sharing statement

Data are available in a public, open access repository. The data underlying the results presented in the study are available from The DHS Program at https://dhsprogram.com/data/new-userregistration.cfm. Data are accessible free of charge upon a registration with the Demographic and Health Survey programme (The DHS Programme).

## Editor note

The Lancet Group takes a neutral position with respect to territorial claims in published maps and institutional affiliations.

## Appendix Supplementary materials

Image, application 1
