# Supplementary material for: Geographical and socioeconomic inequalities in the double burden of malnutrition among women in Southeast Asia: A population-based study
Source: Lancet Reg Health Southeast Asia. 2022 May 23;1:100007. doi: 10.1016/j.lansea.2022.04.003 (PMC10305935; doi:10.1016/j.lansea.2022.04.003)
Supplement: Supplementary file 1 [file mmc1.pdf]

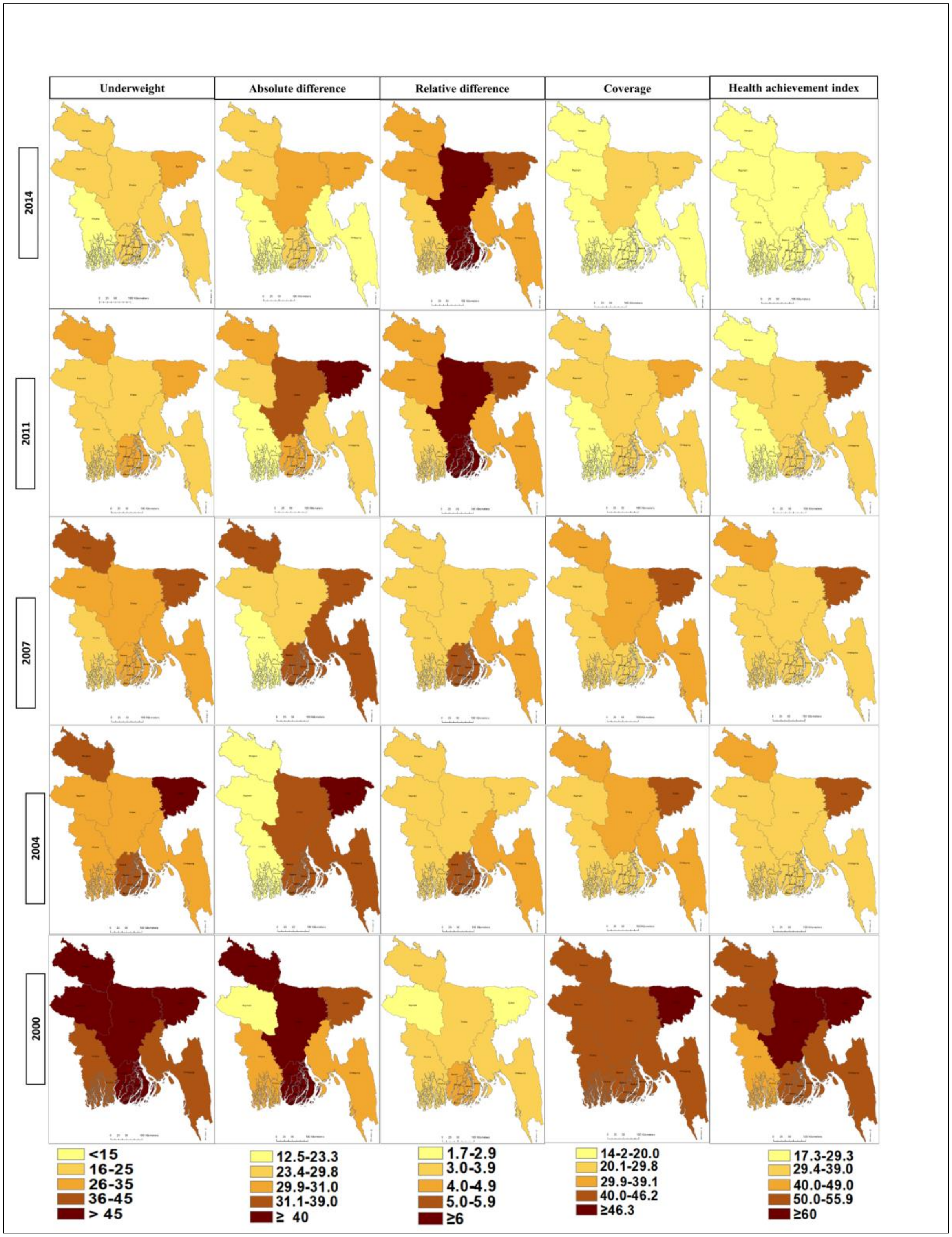

Supplementary figure 1.1: Geographical and socioeconomic inequalities in the women underweight in Bangladesh, 2000-2014

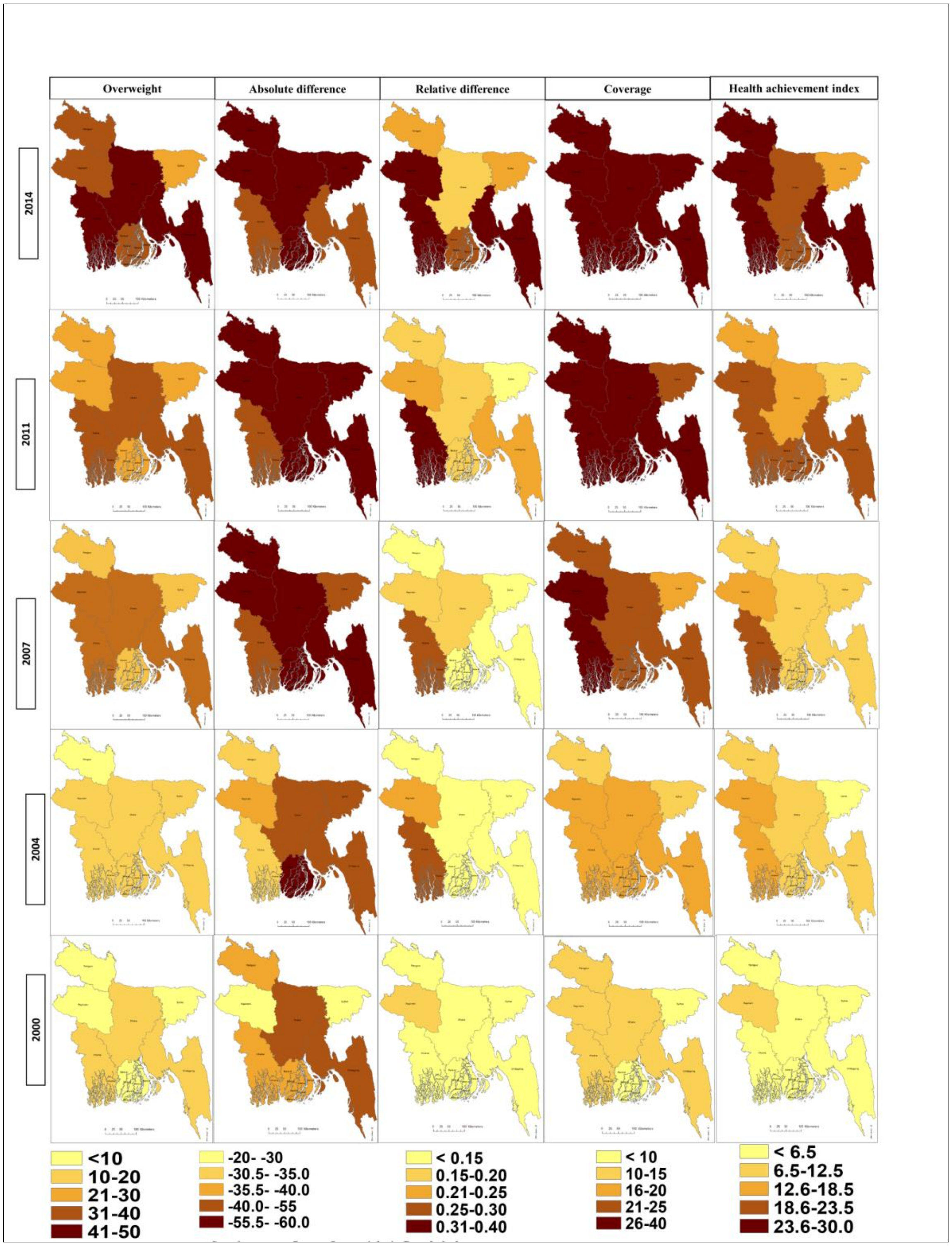

Supplementary figure 1.2: Geographical and socioeconomic inequalities in the women overweight in Bangladesh, 2000-2014

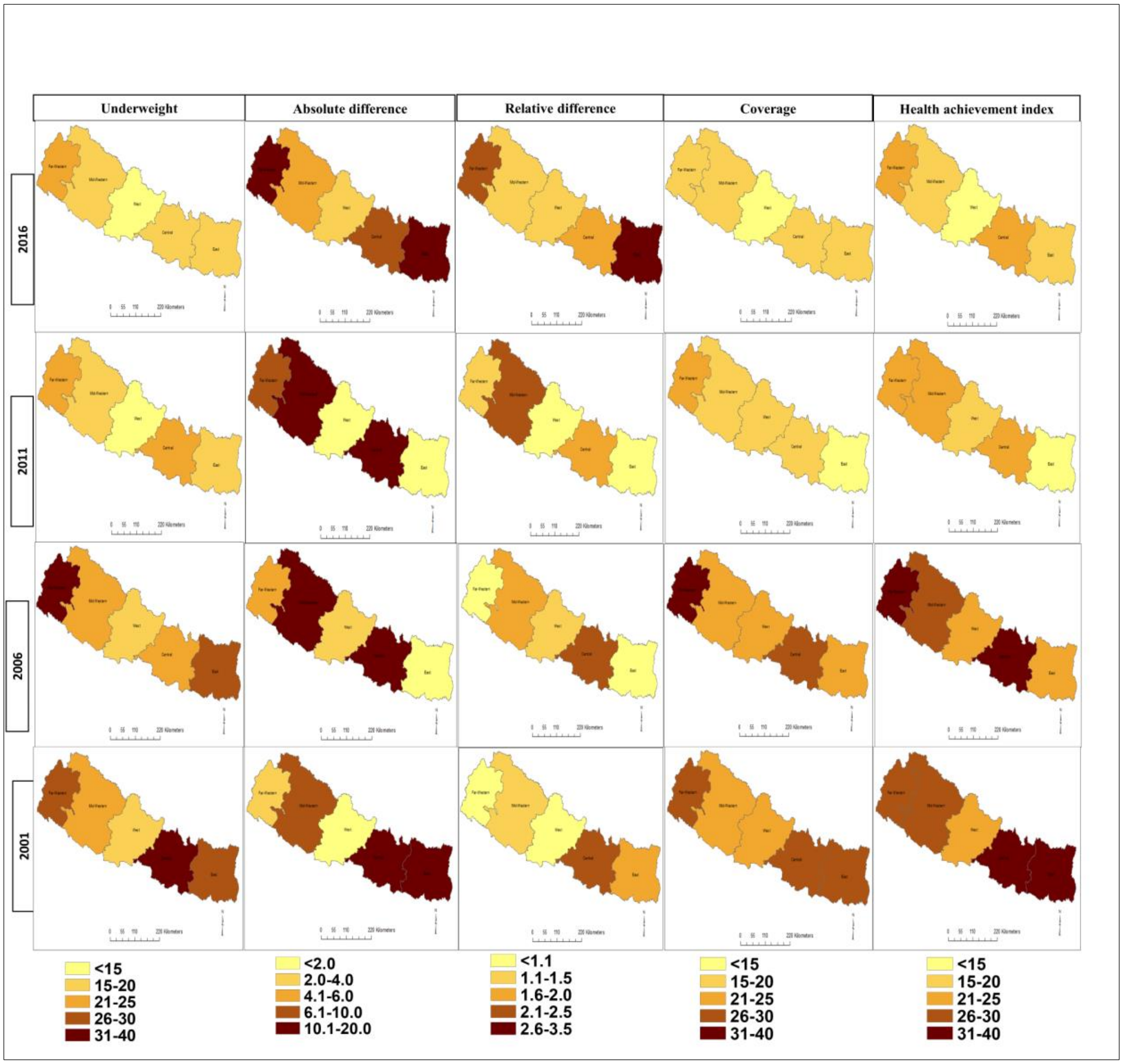

Supplementary figure 21: Geographical and socioeconomic inequalities in the women underweight in Nepal, 2001-2016

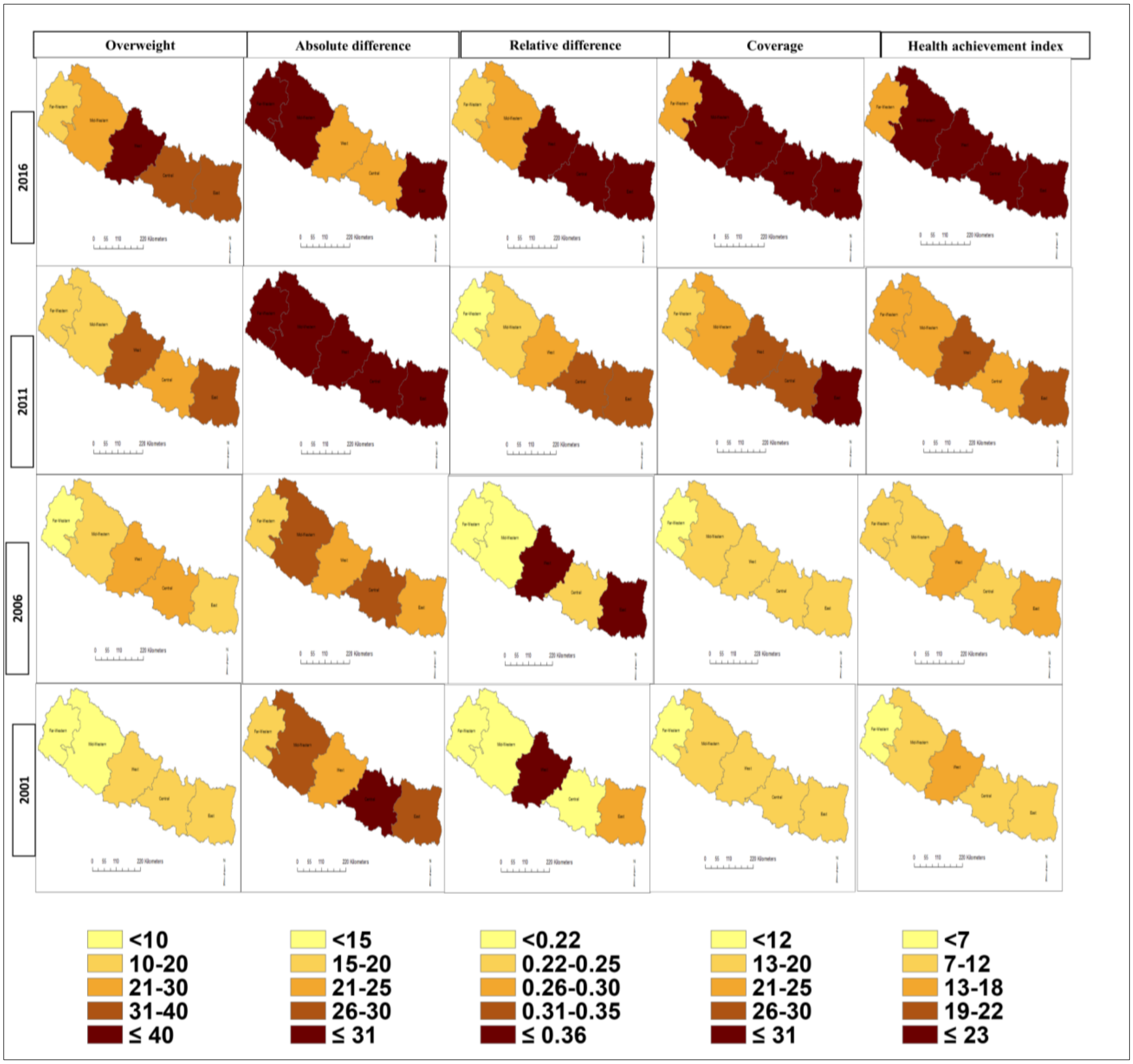

Supplementary figure 2.2: Geographical and socioeconomic inequalities in the women overweight in Nepal, 2001-2016

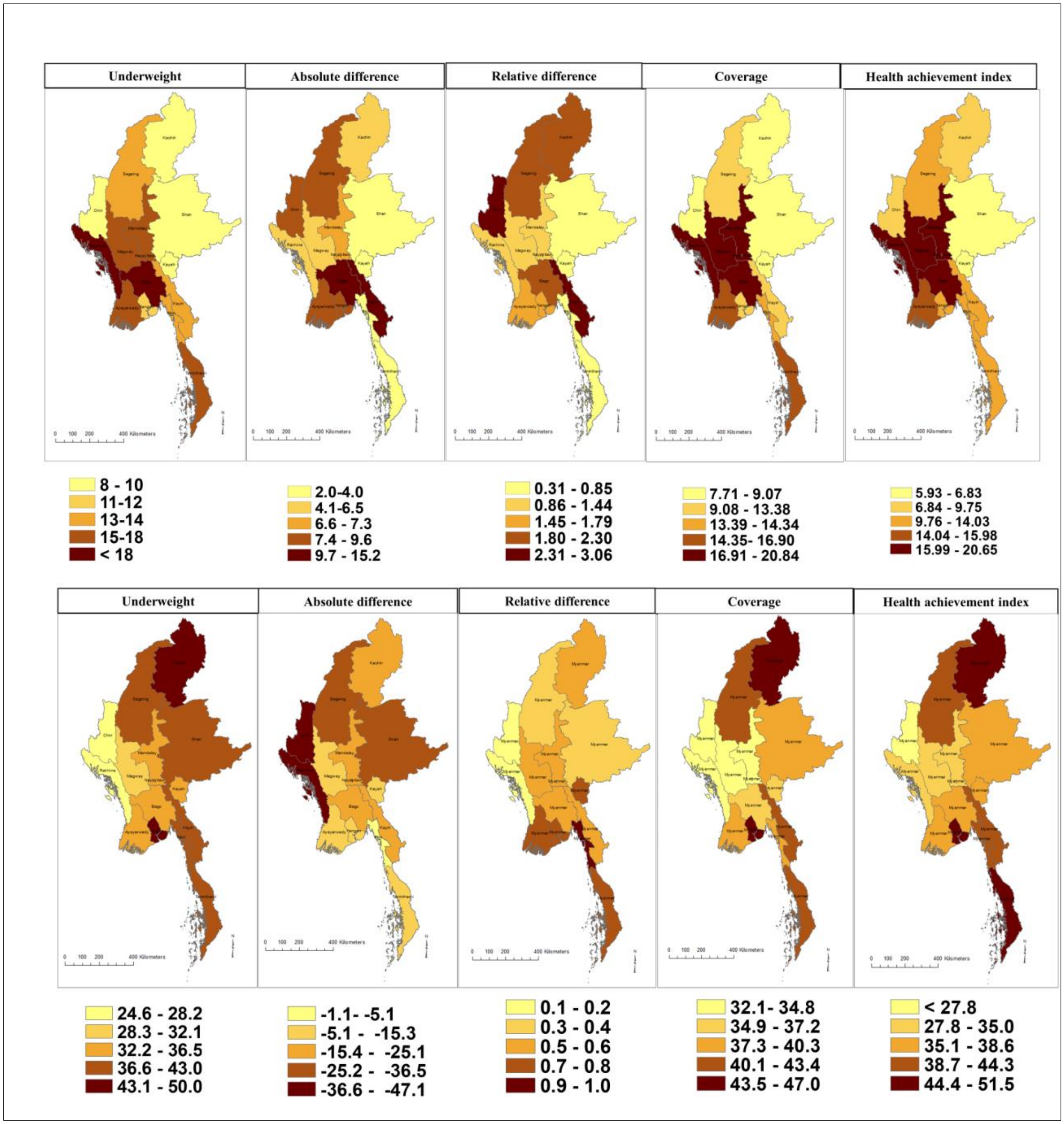

Supplementary figure 3: Geographical and socioeconomic inequalities in the women underweight and overweight in Myanmar

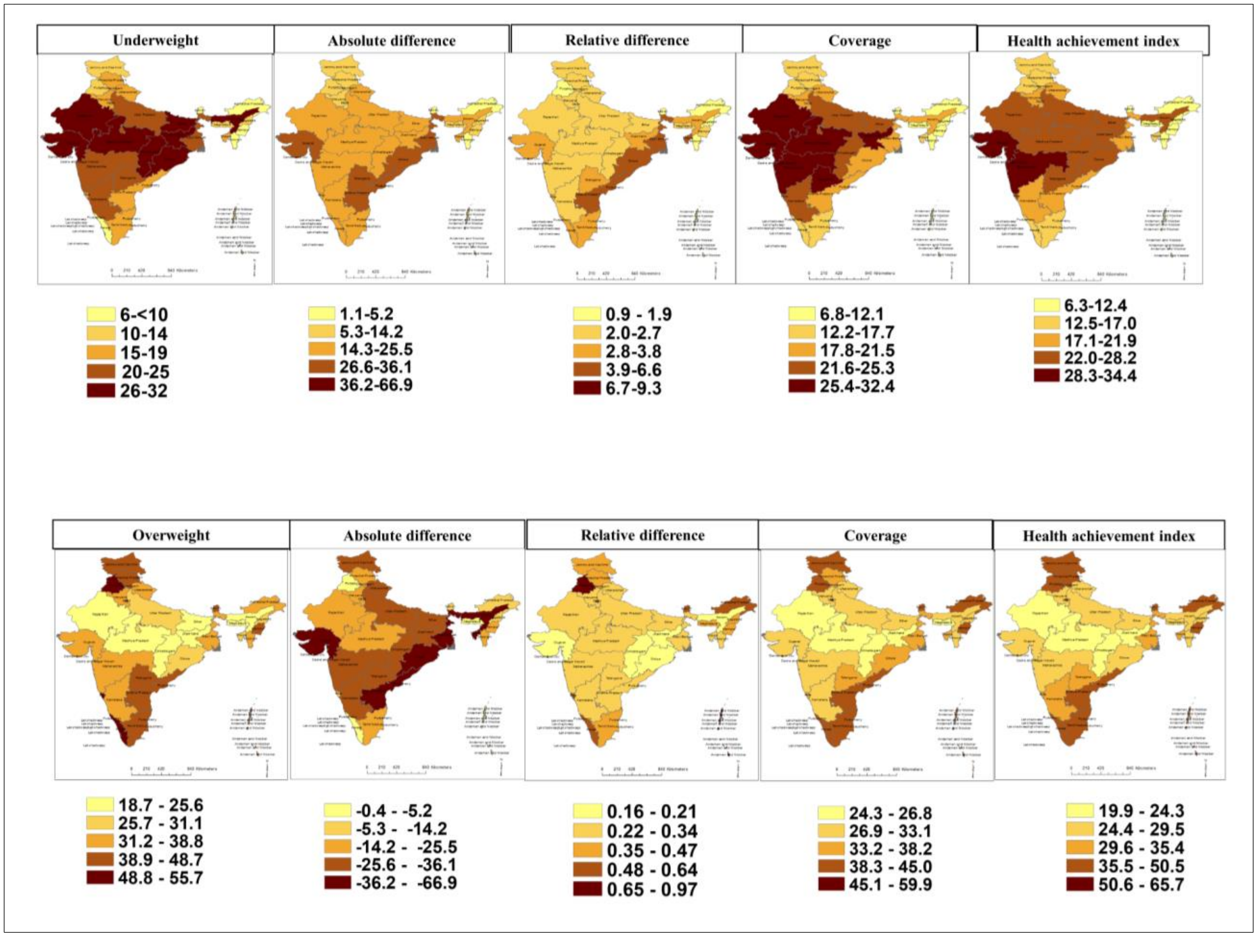

Supplementary figure 4: **Geographical and socioeconomic inequalities in the women underweight and overweight in India**

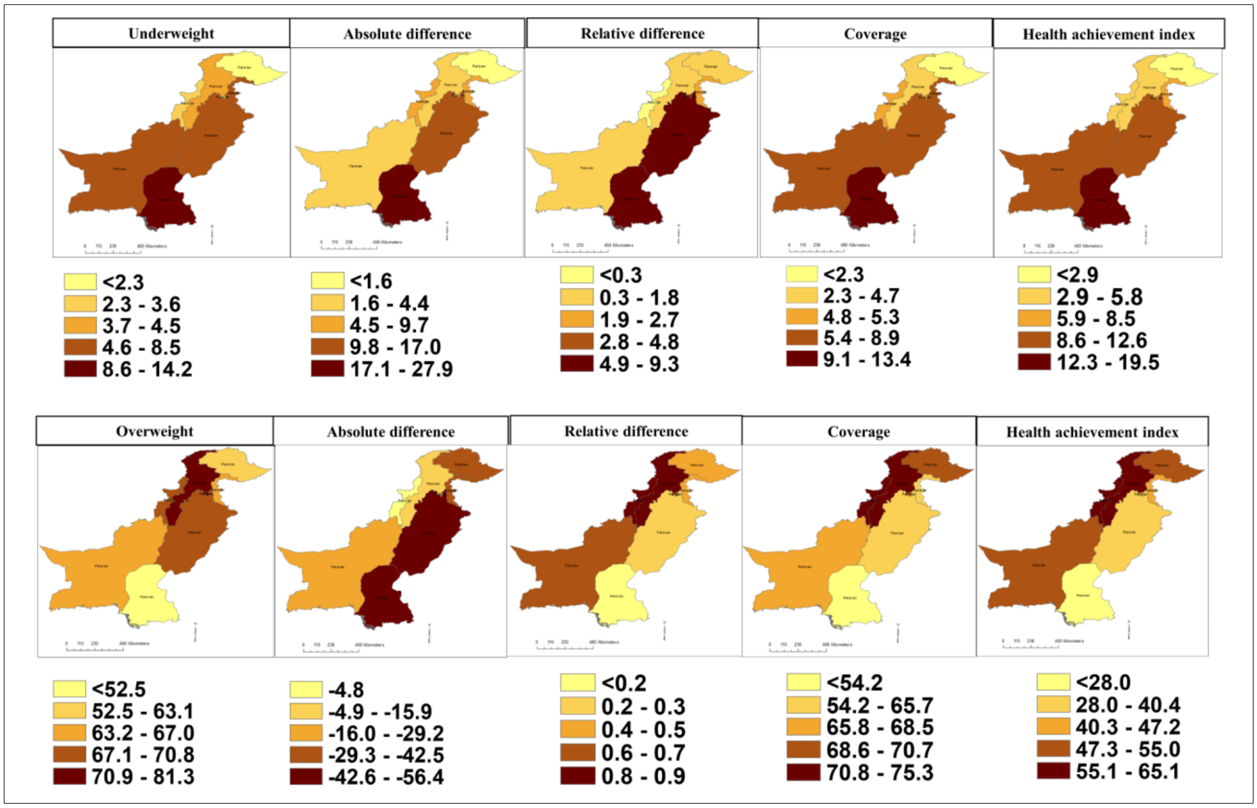

Supplementary figure 5: Geographical and socioeconomic inequalities in the women underweight and overweight in Pakistan

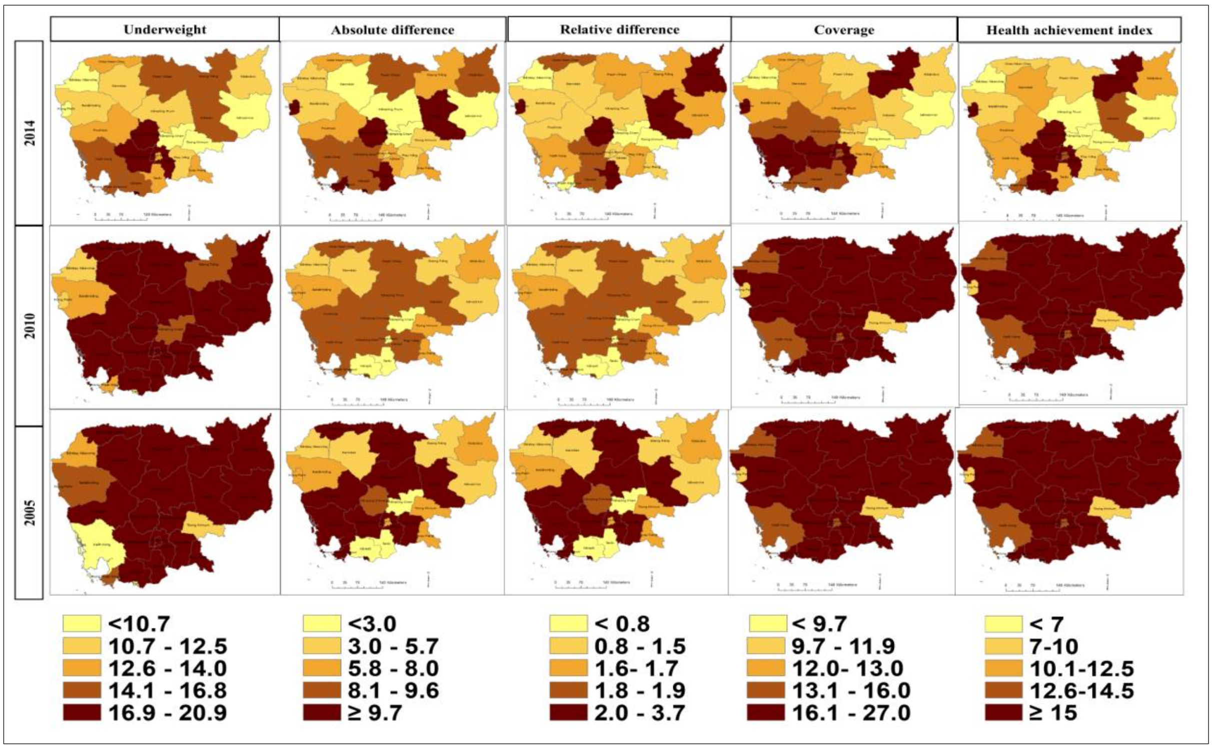

Supplementary figure 6.1: **Geographical and socioeconomic inequalities in the women underweight in Cambodia**

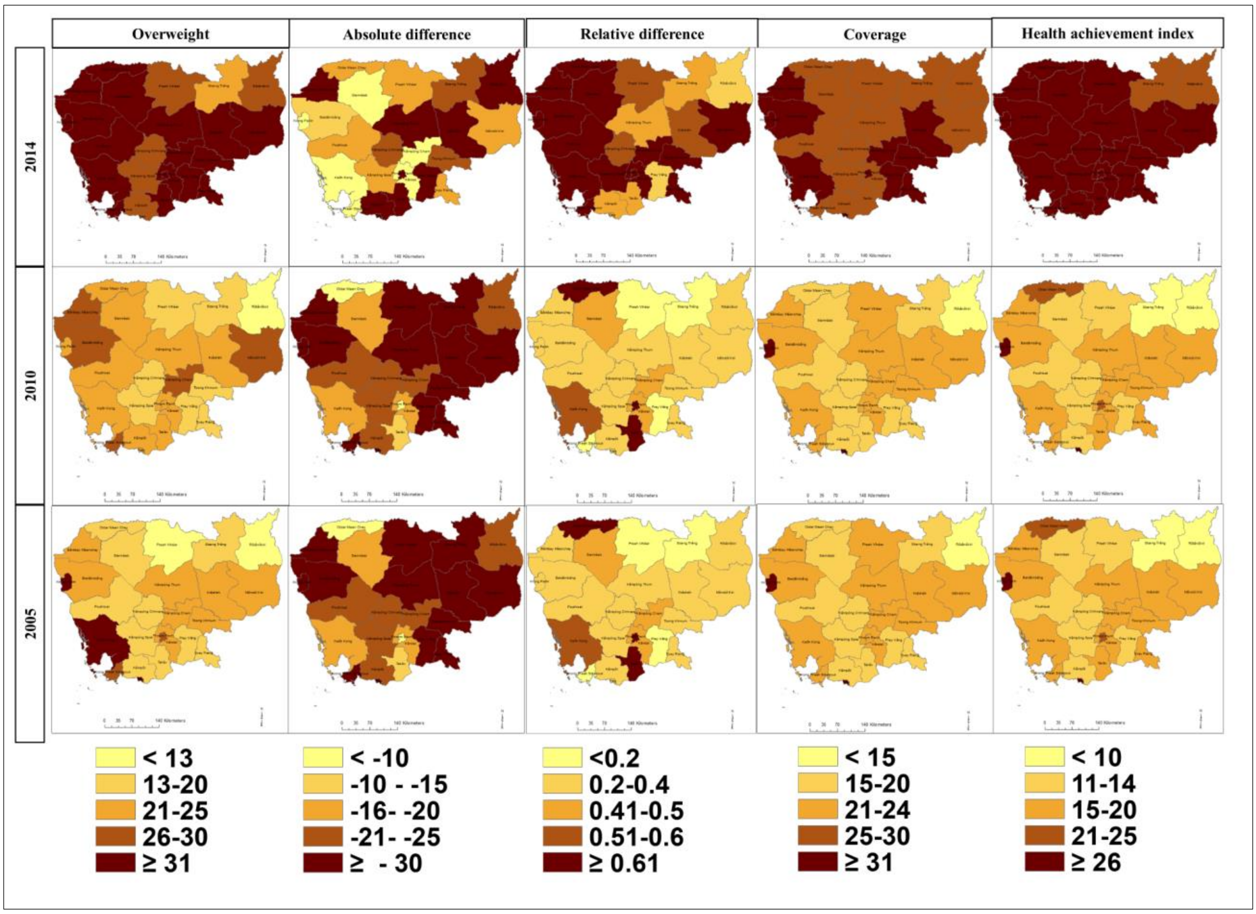

Supplementary figure 6.2: **Geographical and socioeconomic inequalities in the women overweight in Cambodia**

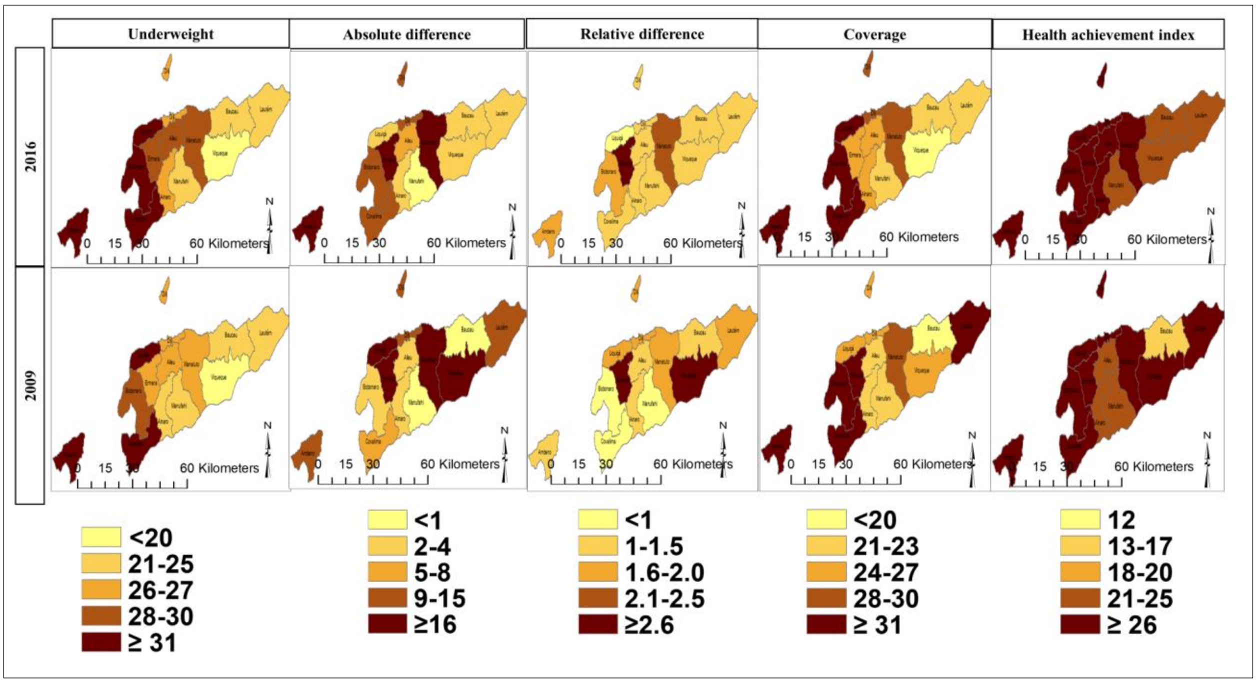

Supplementary figure 7.1: **Geographical and socioeconomic inequalities in the women underweight in Timor**

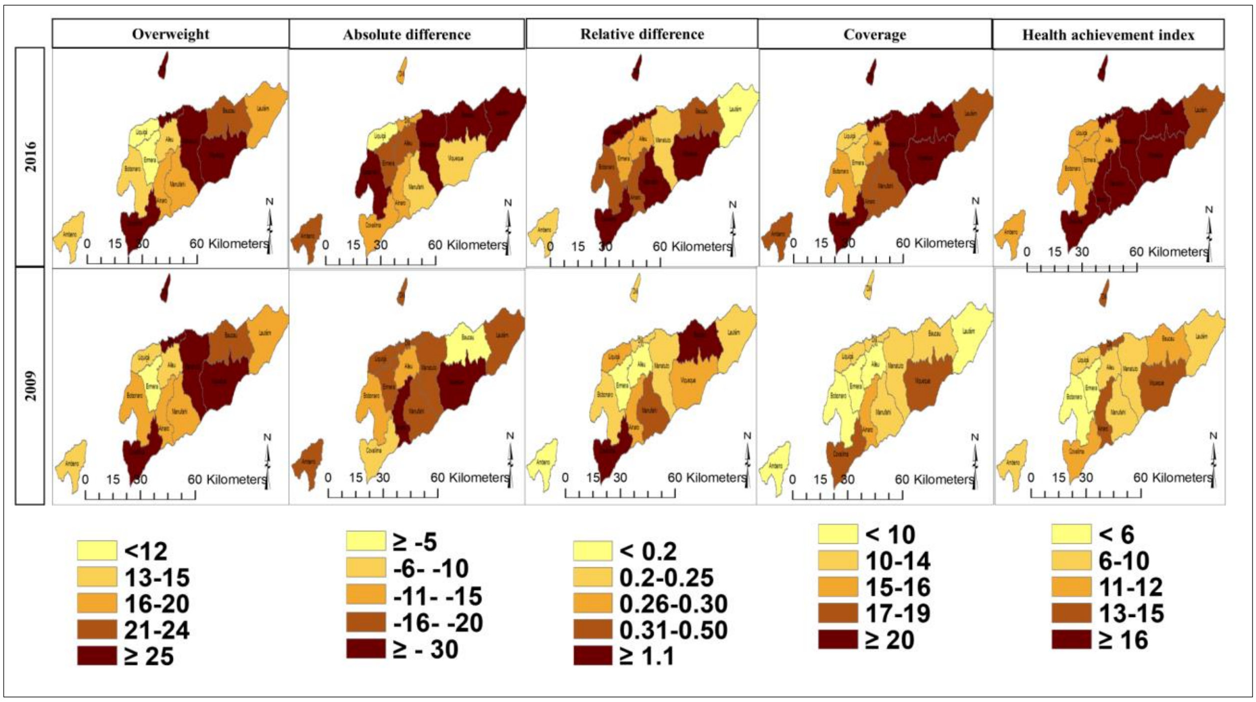

Supplementary figure 7.2: **Geographical and socioeconomic inequalities in the women overweight in Timor**
